# Percutaneous ultrasound-guided renal biopsy: A Libyan experience

**DOI:** 10.4103/0971-4065.65299

**Published:** 2010-04

**Authors:** A. Mishra, R. Tarsin, B. ElHabbash, N. Zagan, R. Markus, S. Drebeka, K. AbdElmola, T. Shawish, A. Shebani, T. AbdElmola, A. ElUsta, E. F. Ehtuish

**Affiliations:** Department of Radiology, National Organ Transplant Center, Central Hospital, Tripoli, Libya; 1 Department of Rheumatology, Tripoli Medical Center, University of AlFateh, Tripoli, Libya; 2 Department of Histopathology, Tripoli Medical Center, Tripoli, Libya; 3 Zawia Clinical Laboratory, Tripoli, Libya; 4 Department of General Surgery, Central Hospital, AlFateh University, Tripoli, Libya; 5 Department of Nephrology, National Organ Transplant Center, Tripoli, Libya; 6 Department of Surgery and Transplant, Central Hospital, University of AlFateh, Tripoli, Libya

**Keywords:** Automated biopsy gun, complications, kidney, percutaneous renal biopsy

## Abstract

This study was done to assess the safety and efficacy of ultrasound-guided percutaneous renal biopsy (PRB), to ascertain the risk factors for complications and determine the optimal period of observation. The radiologist (A.M.) at the National Organ Transplant Centre, Central Hospital, Tripoli, Libya, performed 86 PRBs between February 1, 2006, and January 31, 2008, using an automated biopsy gun with 16-gauge needle. Coagulation profile was done in all the patients. All patients were kept on strict bed rest for six hours post-procedure. Eighty six renal biopsies were performed on 78 patients referred from rheumatology department and eight post-kidney transplant recipients; 23 were males with age range 15 – 56 years and 63 females with age range 16 – 66 years. A mean of 17.5 glomeruli were present in each specimen. A glomerular yield of less than five glomeruli was seen in four biopsies. Class I lupus nephritis (LN) was seen in 1 patient, class II lupus nephritis in 7 patients, class III LN in 13 patients and class IV LN in 29 patients. All the eight renal allografts were diagnosed as acute tubular necrosis or acute interstitial rejection. The risk of post-biopsy bleeding was higher in women, older patients and higher PTT. The overall complication rate was 5.8%. Three complications were observed within six hours of biopsy. No late complication was seen. PRB under real-time ultrasound-guidance is a safe and efficacious procedure to establish the histological diagnosis and should be done as out-patient procedure. Observation time of six hours post-biopsy is optimal.

## Introduction

Percutaneous renal biopsy (PRB) under real-time ultrasound guidance is a routine procedure and allows a histological diagnosis with evidence of renal disease. PRB plays a fundamental role in clinical practice providing important information for diagnosis and prognosis of renal diseases. Like every invasive procedure, renal biopsy is fraught with potential complications. However, with the introduction of automated biopsy guns and real-time ultrasound guidance, the risk of complications has been dramatically reduced. The first PRB was reported in 1951.[1] Renal tissue was obtained by use of a manual technique with a large bore cutting needle.[2] The introduction of automated biopsy devices and the localization of the kidney by ultrasound were aimed at optimizing efficacy and safety of the PRB procedure. We have performed 86 ultrasound-guided PRB with 78 in suspected rheumatologic disease and eight in post kidney transplant patients. On the basis of our experience, we now use the 16-gauge automated biopsy gun under real-time ultrasound guidance as the sole method to obtain core tissue samples from the kidneys for histologic diagnosis of renal parenchymal disease.

## Materials and Methods

### Patients and design

This is a prospective study. All PRBs were performed at the National Organ Transplant Center, Central Hospital, Tripoli, Libya between February 1, 2006 and January 31, 2008. All the biopsies were performed by one radiologist (A.M.) in concordance with the nephrologists or rheumatologists. An informed consent was mandatory in all patients.

### Biopsy procedure

During a two-year period, 86 ultrasound-guided PRBs were performed. Each biopsy was performed with an automated biopsy gun with a 16-gauge needle (C Rose Bard Inc., Murray Hill, NJ). An ATL HDI 5000 ultrasound machine (Philips Medical Systems, Netherlands) was used for localization of the lower pole of the kidney. The length of cutting edge of biopsy gun was 1.8 cm.

The procedure was performed as an out-patient procedure in the Radiology department. Coagulation profile including PT, PTT, and INR, bleeding time, clotting time and total platelet count were done in all the patients. Patients with INR >1.5 or total platelet count < 50 × 10^3^ /ml. were not biopsied.

All patients received local anesthesia prior to the procedure. Pediatric patients also received mild sedation. The patient was placed in prone position for biopsy from native kidney and in supine or decubitus position in posttransplant cases. The kidneys were scanned to determine the optimal biopsy site. The preferred site was the lateral aspect of lower pole of right kidney. In most cases the approach was lateral to medial with a 15° angle of the biopsy gun. The area was prepared and local anesthesia administered. The biopsy gun needle was inserted under ultrasound guidance into the abdominal wall but not pierced into the renal capsule. Then the patient was asked to hold breath after deep inspiration. The needle was then advanced until the tip was seen within the outer cortex. The gun was fired to take the core specimen.

It was the sole discretion of the radiologist performing the procedure to decide the number of “passes” i.e. needle insertions. The radiologist performing the biopsy estimated the number of core samples needed to obtain an adequate specimen, based on visual inspection of each core. The stereomicroscope or a renal pathologist was not available at the site and hence none of the core specimens could be examined immediately for adequate glomerular yield. All patients were kept on strict bed rest for six-hours post-procedure and at least one post-procedure ultrasound scan was performed prior to discharging the patient. The post-procedure ultrasound was done, immediately after the procedure, after six hours of biopsy before discharge, after 24 hours and after two weeks. Post-procedure complications that required surgical intervention or blood transfusion were labeled as ‘major’. ‘Minor’ complications like local pain were managed symptomatically. The patients were followedup by ultrasound and urine examination for two weeks post-biopsy.

The glomerular yield was defined as the total number of glomeruli present in the specimen as observed and reported by a trained renal pathologist. This included the number in the samples for light microscopy (determined by serial sectioning as needed), immunofluorescence and electron microscopy.

All data, results and statistics were compiled and analyzed using statistical software SPSS 11.0 (SPSS Inc., Chicago, IL). Predictors of post biopsy bleeding were assessed by multiple linear and multivariate logistic regression analysis.

## Results

A total of 86 renal biopsies were performed on 78 (90.7%) patients referred from rheumatology department and 8 (9.3%) post-kidney transplant recipients. 23 were males (26.7%) with age range 15 – 56 years and 63 females (73.3%) with age range 16 – 66 years.The main indication for renal biopsy was an elevated serum creatinine (>2 mg/dL) in all the patients. Two ‘passes’ were done in all the native kidneys in addition to one renal allograft and single ‘pass’ in seven renal allografts. The radiologist’s estimate of the number of core samples needed concurred with histopathologist’s determination of sample adequacy in 93% of cases. A mean of 17.5 glomeruli were present in each specimen. A glomerular yield of less than five glomeruli was seen in 4 biopsies. The core sample was reported as ‘inadequate for diagnoses’ in two patients and ‘normal’ in three. Class I lupus nephritis was seen in one patient, class II lupus nephritis in seven patients, class III lupus nephritis in 13 patients and class IV lupus nephritis in 29 patients. Other diagnosis including focal mesangial proliferation, focal sclerosing glomerulosclerosis, chronic glomerulonephritis, mesangiocapillary glomerulonephritis, Fabrys’ disease were seen in 23 (26.7%) patients.

All the eight renal allografts were diagnosed as acute tubular necrosis. All the patients were screened by ultrasound immediately after the procedure, after six hours, 24 hours and two weeks post-procedure. Local biopsy site pain was the most common ‘minor’ complication seen in native kidney biopsies only. The risk of post biopsy bleeding was higher in women, older patients and higher PTT. The overall complication rate was 5.8%. Major complication occurred in one patient leading to loss of renal allograft. Minor complications in form of small perinephric hematoma were noted in two patients with native kidneys. Macroscopic hematuria was seen in two renal allografts of which one developed urinary retention and required intervention while the other was self-limiting. All the three complications were observed within six hours of biopsy. No late complications were seen in any of the patients. There was no difference in the rate of detection of patients with complications after six hours and 24 hours observation. Elective native biopsies were significantly more likely to be associated with pain (*P* = 0.02).

## Discussion

PRB can be fraught with severe complications that may result in loss of kidney and rarely, even death.[3,4] Selection of patients plays a crucial role in avoiding complications. Prior to the procedure, it is imperative to evaluate the patient for history of bleeding diathesis, recent NSAID use, hypertension control, ability to comply with instructions during biopsy and recent pyelonephritis or skin infections near biopsy site.[5] Prebiopsy laboratory tests might include complete blood count and PT/INR, but bleeding time is optional.[6] Stiles *et al*,[7] reported complications in 112 renal biopsies without preceding bleeding times and concluded that the use of bleeding time does not significantly alter the major complication rates. Once a biopsy is scheduled, careful technique and selection of instrumentation contribute to a successful procedure. Since 1990, a safe and reliable renal biopsy technique uses real-time ultrasound guidance with a semiautomated spring-loaded needle.[8]

For patients with difficult landmarks and poor visualization on ultrasound, alternative methods include CT-guidance, transvenous, laparoscopic and open kidney biopsies.[9,10,11] Burstein *et al*,[12] reported complications in 14.3% of 91 patients out of which 6.6% were minor (macrohematuria not requiring transfusion) and 7.7% were major. Mendelssohn and Cole[13] found an overall complication rate of 5.3% in 544 consecutive PRB. Transient gross hematuria occurred in 4.4% of their patients as opposed to 1.2% in the present series. It may be due to the fact that authors did not consistently biopsy with real time ultrasound control.

Our data suggest that native biopsies are likely to be associated with pain requiring analgesia. It is possible that differences between patients may have led to the differing requirements for pain relief. Patients who have renal transplants have previously had numerous surgical procedures including the renal transplant itself and therefore they may have a higher pain threshold. As opposed to this, patients undergoing elective native renal biopsy have no such experience and therefore may have a lower pain threshold. It is possible though that the native kidney with its own nervous innervations is more painful as compared to a transplanted kidney. The present study provides sufficient evidence to allow for the change of practice to perform renal biopsies as a day-case procedure. We have shown that there would have been no difference in the rate of detection of patients with complications if they had been observed for six hours instead of 24 hours post-biopsy.

Chan *et al*,[14] performed PRB on 25 native kidneys and 70 allografts using a 16-gauge automated core biopsy device under real time ultrasound guidance. They concluded that real-time ultrasound guidance in conjunction with an automated core biopsy device is a safe and accurate method of performing PRB in hands of radiologists and they are accurate in estimating sample adequacy in most cases. Manno *et al*,[15] prospectively evaluated the predictive value of demographics, clinical data, baseline chemistry, and needle size for the risk of post-renal biopsy complications in 471 patients. They concluded that only gender, age and baseline partial thromboplastin time show a significant predictive value and the other variables investigated do not have any predictive value. Marwah *et al*,[16] performed PRB in 394 native kidneys and concluded that observation of patients for 23-24 hours is optimal and that observation for 8 hours or less risks missed > or = 20% of complications. On the contrary, our study has shown that observation period of six hours is optimal and that we did not encounter any missed late complications after 24 hours and two weeks of follow-up. Hergesell *et al*,[17] retrospectively analyzed the results of 1090 PRBs and found that ultrasound-guided PRB is a safe procedure and skilled operators obtain satisfactory amounts of kidney tissue in almost all cases. In our study, we had an adequate glomerular yield in 93% of biopsies despite the fact that we did not have the renal pathologist at the site to check for sample adequacy. The major limitation of the study is the small sample size.

## Conclusion

Real-time sonographic guidance in conjunction with an automated 16-gauge core biopsy system is a safe and accurate method in hands of trained and experienced personnel to perform percutaneous renal biopsy and can be safely performed as an out-patient procedure. Observation of patients for at least six hours post-biopsy is optimal.
